# Evaluating the Impact of Human Amnion Epithelial Cells on Angiogenesis

**DOI:** 10.1155/2016/4565612

**Published:** 2015-12-29

**Authors:** Dandan Zhu, Ruth Muljadi, Siow Teng Chan, Patricia Vosdoganes, Camden Lo, Joanne C. Mockler, Euan M. Wallace, Rebecca Lim

**Affiliations:** ^1^The Ritchie Centre, Hudson Institute of Medical Research, Monash University, Clayton, VIC, Australia; ^2^Department of Obstetrics and Gynaecology, Monash University, Clayton, VIC, Australia; ^3^Monash Microimaging, Monash University, Clayton, VIC, Australia

## Abstract

The effects of human amnion epithelial cells (hAECs) on angiogenesis remain controversial. It is yet unknown if the presence of inflammation and/or gestational age of hAEC donors have an impact on angiogenesis. In this study, we examined the differences between term and preterm hAECs on angiogenesis* in vitro* and* in vivo*. Conditioned media from term hAECs induced the formation of longer huVEC tubules on Matrigel. Both term and preterm hAECs expressed* VEGFA*,* PDGFB, ANGPT1,* and* FOXC1*, which significantly increased after TNF*α* and IFN*γ* stimulation. In the presence of TNF*α* and IFN*γ*, coculture with term hAECs reduced gene transcription of* Tie-2* and* Foxc1* in huVECs, while coculture with preterm hAECs increased gene transcription of* PDGFRα* and* PDGFRβ* and reduced gene transcription of* FOXC1* in huVECs.* In vivo* assessment of angiogenesis using vWF immunostaining revealed that hAEC treatment decreased angiogenesis in a bleomycin model of lung fibrosis but increased angiogenesis in a neonatal model of hyperoxia-induced lung injury. In summary, our findings suggested that the impact of hAECs on angiogenesis may be influenced by the presence of inflammation and underlying pathology.

## 1. Background

Human amnion epithelial cells (hAECs) isolated from the amniotic membrane are an attractive source of cell therapy. In addition to their anti-inflammatory and antifibrotic effects, they are nontumorigenic and they exhibit low antigenicity and multipotent differentiation potential [1–3]. Furthermore, they can be isolated in large numbers adequate for clinical applications without requiring serial expansion [2]. We previously applied hAECs isolated from term pregnancies to a mouse model of bleomycin-induced lung fibrosis. We showed that hAEC administration prevented lung inflammation and fibrosis and prevented decline in lung function [4]. In studies, we have also shown that term hAECs can reduce markers of lung inflammation and mitigate structural damage when administered following lung injury induced by either hyperoxia [5] or ventilation [6]. Furthermore, studies on the therapeutic effects of hAECs showed that the engraftment of amnion cells is very rare [5, 7], and hAEC-conditioned media have been shown to contain soluble factors that exert profound biological effects [8]. These studies indicate that hAECs may exert their function in a paracrine fashion.

Given that angiogenesis plays a critical role in wound healing and resolution of inflammation [9, 10], it is important to assess the contribution of angiogenesis to hAEC-augmented repair. There have been contradictory reports on the angiogenic effects of hAECs to date. Specifically, hAECs have been reported to secrete several angiogenic factors* in vitro*. These include tissue inhibitors of metalloproteinases- (TIMP-) 1 and 2, epidermal growth factor (EGF), angiogenin, vascular endothelial growth factor (VEGF), platelet derived growth factor B (PDGFB), and angiogenin [11]. However, when functionally assessed in a rodent dorsal skinfold chamber model hAECs did not increase vessel lengths or vessel sprouts number [12]. It is possible that the effect of hAECs on angiogenesis is altered in an inflammatory environment. Indeed, when administered to a bleomycin-induced lung injury model a week following bleomycin administration when lung inflammation was at its peak, hAECs were unable to mitigate injury [3].

We have also shown that preterm hAECs exert significantly less protective effects* in vivo* compared to term hAECs [13]. This suggests that there may be functional differences between term and preterm hAECs. As such, we sought to assess potential differences in the angiogenic effects of hAECs isolated from different gestational ages. Furthermore, angiogenesis can be either beneficial or detrimental to wound healing, depending on the disease context. Accordingly, we sought to assess the impact of hAECs on angiogenesis using two models of lung injury. The first is a bleomycin-induced lung fibrosis model where angiogenesis is detrimental to the outcome, and the second is a hyperoxia-induced lung injury model where angiogenesis is beneficial.

## 2. Materials and Methods

### 2.1. huVEC and hAEC Isolation

Human umbilical vein endothelial cells (huVECs) were isolated from healthy term human umbilical cords and hAECs were isolated from amnion collected from women undergoing a caesarean section preterm (<37 weeks' gestation) or at term as previously described [2, 14]. Preterm donors included women delivering prematurely due to preeclampsia, gestational hypertension, fetal growth restriction, placenta praevia, and discordant twin growth. Donors with pregnancies complicated by chorioamnionitis and preexisting maternal diseases were excluded. The term donors were women with a healthy pregnancy undergoing an elective repeat caesarean section. The mean gestational age for preterm birth was 230 ± 7 days and for term birth 268 ± 1 days (Table 1). All collection and isolation procedures were undertaken with the approval from Monash Health Human Ethics Committee and with written informed consent.

### 2.2. Collection of hAEC-Conditioned Media

The collection of hAEC-conditioned media followed previous protocol [15]. Briefly, hAECs were plated at a density of 10 million cells per T175 flask and conditioned media were collected following 96 hours under standard tissue culture conditions.

### 2.3. *In Vitro* Angiogenesis Assay

On a Matrigel (50 *μ*L/well; Corning Life Sciences) coated 96-well plate, 1 × 10^4^ huVECs at passage 3 were seeded in each well. Cells were cultured in either M199 media (Invitrogen) [16] or hAEC-conditioned media. Phase contrast images were taken every 30 minutes for a total of 40 hours. The capillary-like structures were detected and the average length of tubes between branches was calculated using Image J (National Institutes of Health, Bethesda, MA) and Metamorph software (Molecular Devices, Sunnyvale, CA) programs.

### 2.4. Stimulation of hAECs

Preterm (*n* = 6) and term (*n* = 6) hAECs were cultured in the presence or absence of tumor necrosis factor *α* (TNF*α*) (20 ng/mL, PHC3015, Life Technologies) and interferon *γ* (IFN*γ*) (20 ng/mL, PHC4031, Life technologies) for 24 hrs and 48 hrs. Concentrations of TNF*α* and IFN*γ* were based on a previous study by Liu and colleagues who reported an increase in angiogenic potential of MSCs following exposure to inflammatory cytokines [17].

### 2.5. Coculture of huVECs and hAECs

hAECs were seeded at a density of 5 × 10^5^ cells in each well of a 6-well plate, while 1 × 10^5^ huVECs were seeded into each 0.4 *μ*m pore transwell insert (BD Bioscience, San Jose, CA) in M199 media in the presence or absence of TNF*α* and IFN*γ*.

### 2.6. Gene Expression Assays

RNA was isolated using the RNeasy Mini Kit (Qiagen, Limburg, Netherlands) and 1 *μ*g total RNA was converted to cDNA using the Thermoscript Reverse Transcription System (Invitrogen). qRT-PCR was performed using SensiMix SYBR and Rotor-Gene (Qiagen). Primers for hAECs were directed against* VEGFA*,* PDGFB*, angiogenin-1 (*ANGPT1*), and Forkhead box (FOX) transcription factor* FOXC1*. Primers for huVECs were directed against vascular endothelial growth factor receptors 1 and 2 (*VEGFR1 and VEGFR2*), platelet derived growth factor receptors alpha and beta (*PDGFRα and PDGFRβ*),* Tie-2,* and* FOXC1*. Gene expression was normalised to 18S and expressed relative to either unstimulated hAECs or huVECs. Detailed information on primer sequences is listed in Table 2.

### 2.7. Animals and Experimental Groups

All animal experiments were approved by the Monash Medical Centre Animal Ethics Committee and were conducted in accordance with the Australia Code of Practice for Care and Use of Animals for Scientific Purpose (2006). In the bleomycin-induced mouse lung injury model, 6–8-week-old female C57/BL6 mice weighing 16–20 g were housed in a specific pathogen-free animal facility during this study. Experimental groups included saline + saline, saline + term hAECs, bleomycin + saline, bleomycin + term hAECs, and bleomycin + preterm hAECs. The mice were given either saline or 0.3 IU bleomycin (Blenoxane, Hospira, Lake Forest, IL, USA) intranasally, followed by intraperitoneal administration of 4 million term hAECs or preterm hAECs, or 200 *μ*L saline 24 hours later, as previously described [13]. Mice were culled 14 days following intranasal instillation of bleomycin by carbon dioxide asphyxiation. The right lungs were inflated and fixed with 4% (w/v) paraformaldehyde processed for immunofluorescence.

In the neonatal mouse model of hyperoxia-induced lung injury, newborn C57/BL6 mouse pups were randomised to either normoxia (inspired O_2_ content (FiO_2_) = 0.21) or hyperoxia (FiO_2_ = 0.85). Experimental groups included normoxia + saline, normoxia + term hAECs, hyperoxia + saline, and hyperoxia + term hAECs. On postnatal days (PND) 4, 5, and 6, a total of 4.5 million term hAECs or 50 *μ*L sterile saline (control) was administered intraperitoneally as previously described [5]. On postnatal day 14, mice were culled and lungs were collected in 4% PFA prior to processing and embedding in paraffin.

### 2.8. Immunohistochemistry Staining for vWF

Pulmonary vessels were assessed by von Willebrand factor (vWF) staining of paraffin embedded lung tissue sections. The slides were subjected to proteinase K retrieval, followed by incubation with primary antibody polyclonal rabbit anti-human vWF (1 : 400, Dako, A0082, Germany) overnight at 4°C. After washing with PBS, LSAB +/HRP kit (Dako, K0690, Germany) was applied to the sections for 1 hour at room temperature, followed by labelling with streptavidin-horseradish peroxidase and diaminobenzidine (DAB, Dako, K3408, Germany). Nuclei were counterstained with haematoxylin. The percentage of vWF positive area was determined in bleomycin mice by Image J (NIH). In the hyperoxia study, the number of vWF positive vessels with diameter less than 50 *μ*m was counted in 15 random images at 200x magnification using Image J (NIH).

### 2.9. Data Analysis

Data were expressed as mean ± standard error of mean (SEM). Statistical significance was determined using GraphPad Prism (GraphPad Software Inc., San Diego, CA, USA) with one-way ANOVA accompanied by the Bonferroni* post hoc* test for multiple groups or the Mann-Whitney test when comparing between two groups. Statistical significance was accorded when *p* < 0.05.

## 3. Results

### 3.1. Vascular Tube Formation in huVECs on Matrigel

**Figure 1:**
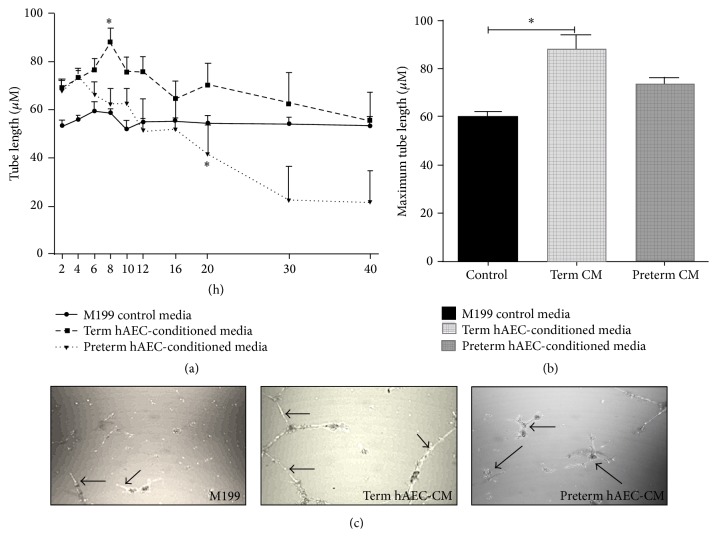
The vascular tube formation in huVECs on Matrigel* in vitro*. ((a) and (b)) huVECs formed longer vascular tubules following culture in term hAEC-conditioned media compared to control media (*p* < 0.05), and tubule lengths were stable for 40 hours. huVECs cultured in preterm hAEC-conditioned media did not support tubule formation where tubule length was significantly reduced at 20 hours (*p* < 0.05). (c) Representative pictures of vascular tubules of huVECs following culture in term hAEC-conditioned media and control media (^*∗*^ *p* < 0.05).

The maximum length of vascular tubes formed by huVECs appeared at 6 hours following culture in term hAEC-conditioned media and M199 media and appeared at 4 hours following culture in preterm hAEC-conditioned media. Changes in average vascular tube lengths over a 40-hour period are depicted in Figure 1(a). The average maximum length of vascular tubes formed in term hAEC-conditioned media was significantly greater compared with M199 control media (Figure 1(b), 88.03 ± 5.77 *μ*m versus 59.76 ± 2.19 *μ*m, *p* < 0.05) but not the preterm hAEC-conditioned media (73.52 ± 2.86 *μ*m), which was not significantly different to either control conditions or term hAEC-conditioned media. On average, the tubules of huVECs cultured in term hAEC-conditioned media and control media were stable for up to 40 hours. In contrast, the average length of huVEC tubules cultured in preterm hAEC-conditioned media decreased after 16 hours, such that by 20 hours they were significantly shorter than tubules cultured in either term hAEC-conditioned media or control media (*p* < 0.05). This suggests that preterm hAEC-conditioned media contain soluble factors that reduce tubule stability. Representative images of huVEC tubules formed following culture in term hAEC-conditioned media and control media are shown in Figure 1(c).

### 3.2. Gene Expression of Angiogenic Ligands by hAECs

**Figure 2:**
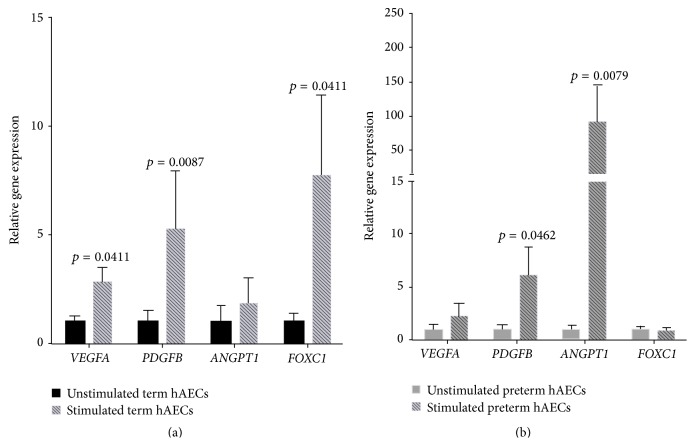
Gene expression of angiogenic ligands by hAECs following stimulation with TNF*α* and IFN*γ*. (a) Gene expressions of* VEGFA* (*p* = 0.0411),* PDGFB* (*p* = 0.0087), and* Foxc1* (*p* = 0.0411) increased in term hAECs. (b) Gene expression of* PDGFB* (*p* = 0.0462) and* ANGPT1* (*p* = 0.0079) increased in preterm hAECs.

We assessed gene transcription of* VEGFA, PDGFB*,* ANGPT1,* and* FOXC1* in term and preterm hAECs under basal conditions and following exposure to TNF*α* and IFN*γ*, which were used to mimic an inflammatory environment. All of these genes were transcribed by both term and preterm hAECs under basal conditions. In term hAECs, stimulation with TNF*α* and IFN*γ* significantly increased transcription of* VEGFA*,* PDGFB,* and* FOXC1* (Figure 2(a), 2.82 ± 0.66, *p* = 0.0411; 5.27 ± 2.68, *p* = 0.0087, and 7.72 ± 3.69, *p* = 0.0411, resp.). Gene transcription of* ANGPT1* remained unchanged. In preterm hAECs, stimulation with TNF*α* and IFN*γ* increased gene transcription of* PDGFB* and* ANGPT1* (Figure 2(b), 6.14 ± 2.73, *p* = 0.0462 and 90.92 ± 55.21, *p* = 0.0079, resp.). Gene transcription of* VEGFA* and* FOXC1* remained unchanged.

### 3.3. Gene Expression of Angiogenic Receptors by huVECs

**Figure 3:**
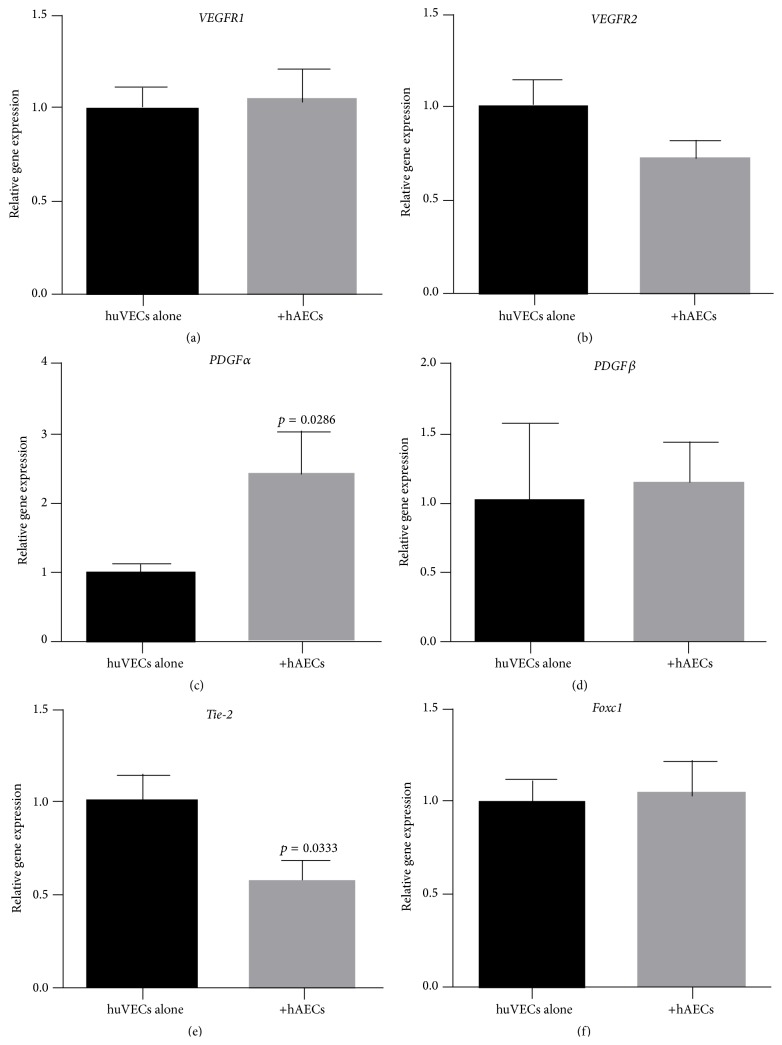
Gene expression of angiogenic factors by huVECs cocultured with term hAECs under basal conditions. ((a)–(f)) huVECs expressed* VEGFR1*,* VEGFR2*,* PDGFRα*,* PDGFRβ*,* Tie-2,* and* FOCX1.* Following coculture with term hAECs, ((c) and (e)) gene expression of* PDGFRα* increased (*p* = 0.0286), but gene expression of* Tie-2* decreased (*p* = 0.0333), ((a), (b), (d), and (f)) while there was no change in gene expression of* VEGFR1*,* VEGFR2*,* PDGFRβ*, and* FOXC1*.

We next assessed the impact of term hAECs on huVECs under basal conditions and in the presence of TNF*α* and IFN*γ*. Under basal conditions, huVECs expressed receptors for pro- and antiangiogenic factors including* VEGFR1*,* VEGFR2*,* PDGFRα*,* PDGFRβ*,* Tie-2,* and* FOXC1* (Figures 3(a)–3(f)). Following coculture with term hAECs, we noted a significant increase in transcription of* PDGFRα* (Figure 3(c), 2.39 ± 0.62 versus 1.0 ± 0.11, *p* = 0.0286) and decrease in* Tie-2* (Figure 3(e), 0.56 ± 0.11 versus 1.0 ± 0.15, *p* = 0.0333).

**Figure 4:**
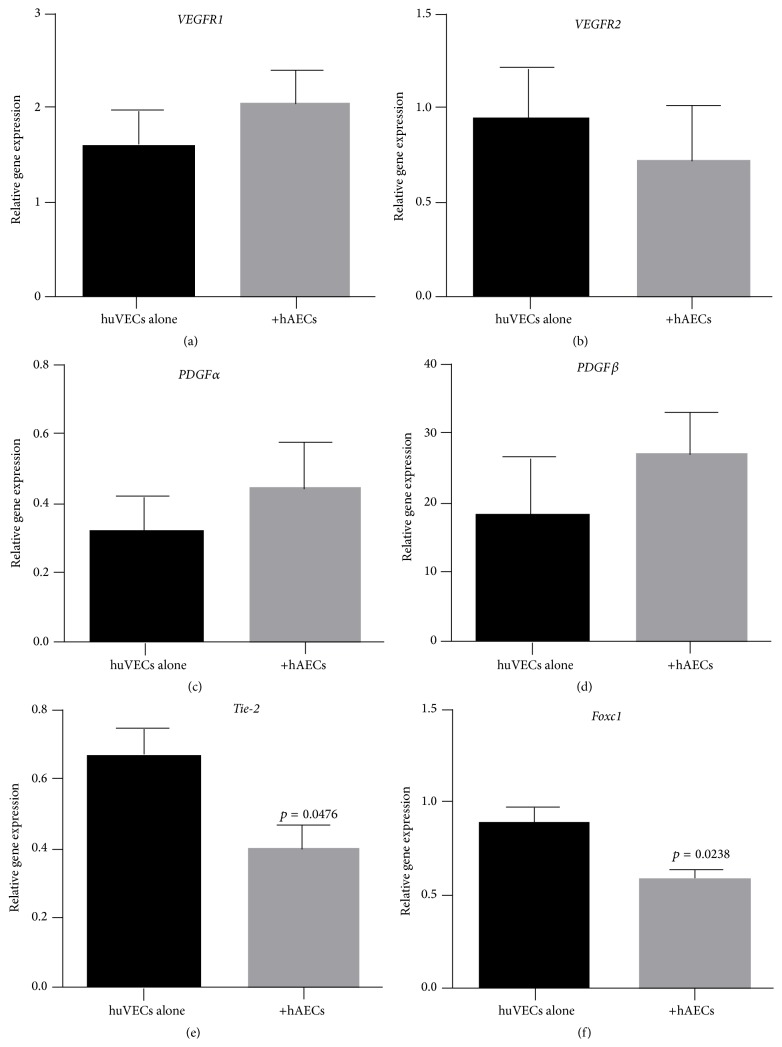
Gene expression of angiogenic factors by huVECs cocultured with term hAECs in the presence of TNF*α* and IFN*γ*. ((a)–(d)) There was no change in gene expression of* VEGFR1*,* VEGFR2*,* PDGFRα*,* and PDGFRβ*, ((e) and (f)) while gene expression of* Tie-2* and* FOXC1* was significantly reduced (*p* = 0.0476 and *p* = 0.0238, resp.).

**Figure 5:**
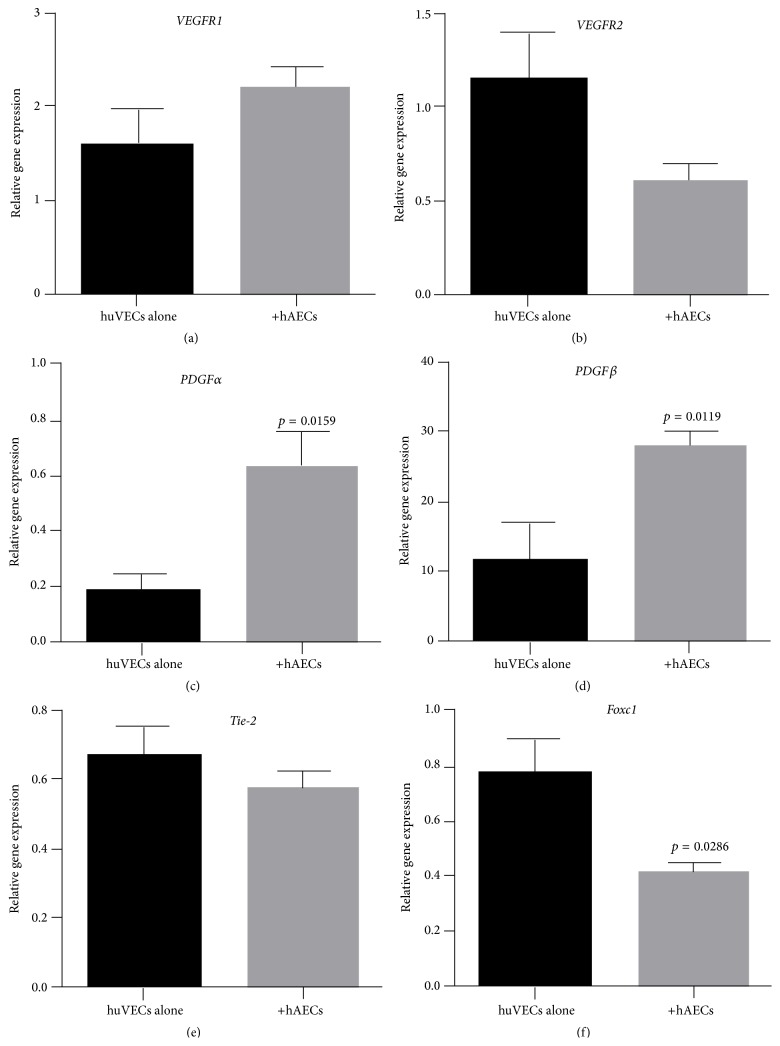
Gene expression of angiogenic factors in huVECs cocultured with preterm hAECs in the presence of TNF*α* and IFN*γ*. ((a), (b), and (e)) There was no change of gene expression of* VEGFR1*,* VEGFR2*,* and Tie-2*. ((c), (d), and (f)) While gene expression of* FOXC1* was also significantly reduced (*p* = 0.0286), gene expression of* PDGFRα* and *β* was significantly increased (*p* = 0.0159 and *p* = 0.0119, resp.).

We next assessed the impact of term and preterm hAECs on huVECs stimulated by TNF*α* and IFN*γ*. When cocultured with stimulated term hAECs, huVECs significantly reduced gene transcription of* Tie-2* (Figure 4(e), 0.40 ± 0.07 versus 0.67 ± 0.08, *p* = 0.0476) and* Foxc1* (Figure 4(f), 0.59 ± 0.04 versus 0.88 ± 0.09, *p* = 0.0238). When cocultured with stimulated preterm hAECs, huVECs significantly increased gene transcription of* PDGFRα* (Figure 5(c), 0.64 ± 0.12 versus 0.18 ± 0.06, *p* = 0.0159) and*β* (Figure 5(d), 27.92 ± 1.96 versus 11.6 ± 5.33, *p* = 0.0119). Gene expression of* Foxc1* was also significantly reduced (Figure 5(f), 0.42 ± 0.03 versus 0.78 ± 0.12, *p* = 0.0286).

### 3.4. Assessment of Angiogenesis* In Vivo*

**Figure 6:**
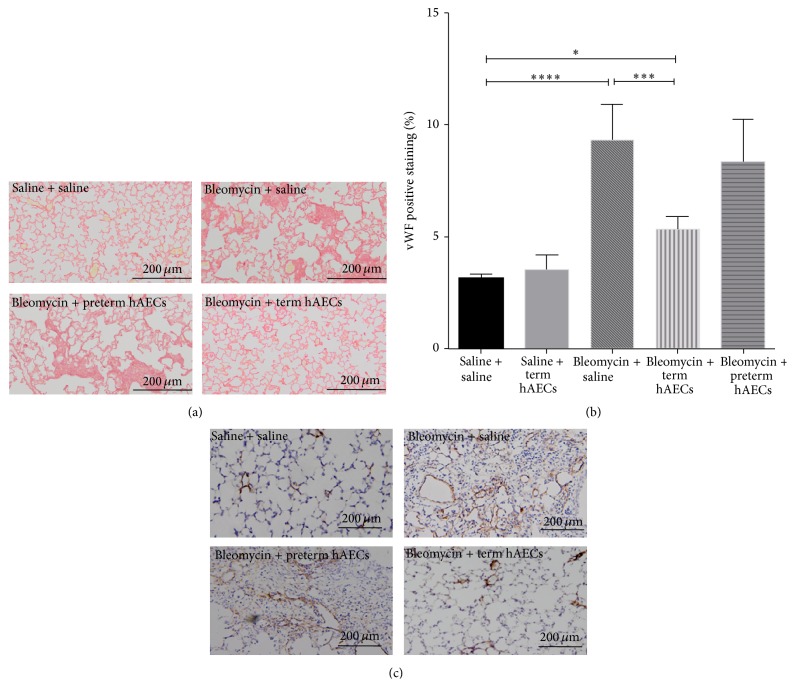
Collagen staining and vWF immunohistochemistry in bleomycin challenged mouse lung tissue. (a) Bleomycin challenged mice had more fibrotic tissues in the lung compared to control group; term hAEC treatment, but not preterm hAEC treatment, reduced the fibrotic tissues. (b) The percentage of vWF positive staining increased in bleomycin-injured animals compared to control group (*p* < 0.0001) and decreased after term (*p* < 0.001) but not preterm hAEC administration. (c) The representative images for vWF immunohistochemistry in mouse lung tissues (^*∗*^ *p* < 0.05, ^*∗∗∗*^ *p* < 0.001, and ^*∗∗∗∗*^ *p* < 0.0001).

Term but not preterm hAEC administration reduced pulmonary fibrosis following bleomycin challenge (Figure 6(a)). The percentage of vWF positive staining increased significantly in the lungs of bleomycin-alone animals (9.31 ± 0.69% versus 3.12 ± 0.47%, *p* < 0.0001). This was mitigated by the administration of term (5.34 ± 0.25%, *p* < 0.001) but not preterm hAECs (Figure 6(b)). Representative images of immunohistochemical staining are shown in Figure 6(c).

**Figure 7:**
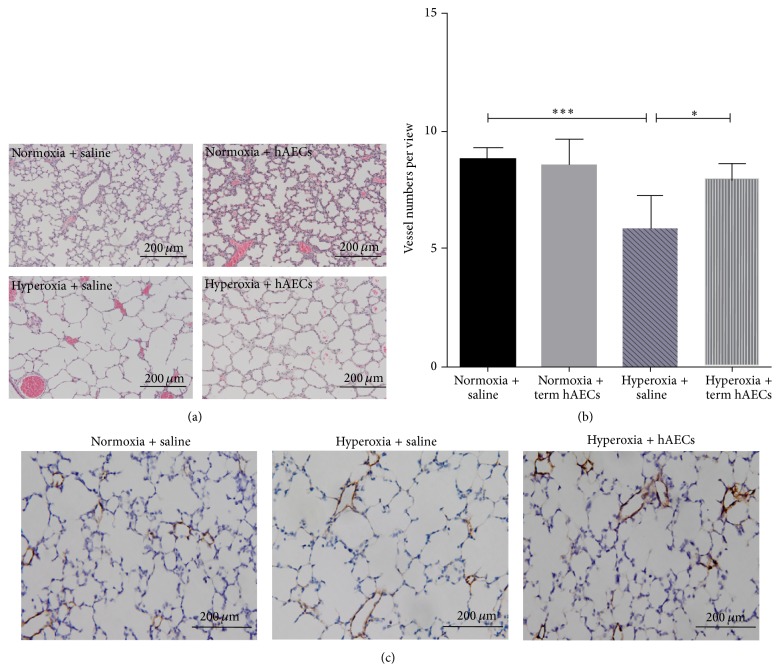
H&E staining and vWF immunohistochemistry in neonatal mouse lung tissue. (a) Hyperoxia-induced lung injury mice had simplified lung structure and enlarged alveoli, and term hAEC treatment improved lung structure. (b) The number of vessels (diameter < 50 *μ*m) decreased in hyperoxia-injured animals compared with normoxia animals (*p* < 0.001). Term hAEC administration restored the number of small pulmonary vessels in hyperoxia-induced lung injury animals (*p* < 0.05). (c) The representative images for vWF immunohistochemistry in mouse lung tissues (^*∗*^ *p* < 0.05, ^*∗∗∗*^ *p* < 0.001).

Given that only term but not preterm hAECs result in an effect in the adult mice, we proceeded to assess the impact of term hAECs on angiogenesis in a hyperoxia-induced lung injury model in neonatal mice. Here we observed that mice in hyperoxia group had simplified lung structure and enlarged alveoli, and term hAEC treatment improved lung structure (Figure 7(a)). The number of small pulmonary vessels (diameter < 50 *μ*m) decreased in hyperoxia-injured neonatal mice compared with normoxia mice (5.84 ± 0.58% versus 8.80 ± 0.21%, *p* < 0.001). Term hAEC administration restored the number of small pulmonary vessels in hyperoxia-induced lung injury animals (7.88 ± 0.33%, *p* < 0.05) (Figure 7(b)). Representative images of immunohistochemical staining are shown in Figure 7(c).

## 4. Discussion

The ability of hAECs to support angiogenesis is poorly understood. Additionally, little is known about the importance of gestational age of hAEC donors to the contribution of angiogenesis during repair. In this study, we showed that* in vitro* tubule formation by huVECs was best supported by term hAECs compared to preterm hAECs. Both term and preterm hAECs transcribed genes of proangiogenic ligands* VEGFA*,* PDGFB, and ANGPT1* and transcription factor,* FOXC1*. These were upregulated by inflammatory cytokines, IFN*γ* and TNF*α*. However, coculture with both term and preterm hAECs did not consistently increase gene transcription of proangiogenic receptor ligands in huVECs. When we assess the effects of hAEC treatment on angiogenesis* in vivo* using a bleomycin model of lung fibrosis, we found that while term hAECs reduced vWF staining in the lungs, consistent with resolution of lung fibrosis, treatment with preterm hAECs had no effect. This observation coincided with our previous study showing that preterm hAECs had diminished reparative effects. When we assess the effects of term hAEC treatment on angiogenesis in a model of hyperoxia-induced neonatal lung injury, we found instead that hAEC treatment was associated with improvement in pathological lung remodelling.

The Matrigel tubule formation assay is an established method for evaluating the angiogenic effects of soluble factors in endothelial cells* in vitro* [18]. Using this assay, we determined that term hAECs release more proangiogenic factors compared to their preterm counterparts, supporting endothelial cell tubule formation as previously reported with bone marrow-derived MSCs [19]. In order to elucidate the nature of these proangiogenic factors, we compared the gene expressions of proangiogenic ligands. Angiogenic factors, such as ANGPT1, PDGFB, and VEGFA, have been previously detected in the secretome of human MSC from different tissue sources [20]. Stimulation by TNF*α* and LPS increased the production of VEGFA by adipose-derived MSCs [21] while transforming growth factor *α* (TGF-*α*) induced the secretion of VEGFA and PDGFB in bone marrow-derived MSCs [22]. Similarly, amnion derived mesenchymal stromal cells (MSCs) secrete angiogenic factors including EGF, VEGF, TIMP-1, and TIMP-2 [11].

In our current study we found that both preterm and term hAECs expressed* VEGFA*,* PDGFB*,* ANGPT1,* and* FOXC1* under basal conditions. We then looked to see if the hAECs altered transcription of these genes in response to proinflammatory stimuli by exposing them to a combination of TNF*α* and IFN*γ*. VEGFA stimulates the generation of new, immature, and leaky blood vessels by disrupting the basement membrane of the preexisting vessels, inducing endothelial cell migration and proliferation [23, 24], while PDGF and ANGPT1 are essential for the stabilisation of new vessels. These angiogenic ligands promote angiogenesis, induce vascular maturation, and decrease vascular permeability by mediating migration, adhesion, and survival of endothelial cells [25]. Interestingly, transcription of* VEGFA*,* PDGFB,* and* FOXC1* was elevated in term hAECs, while* PDGFB* and* ANGPT1* were increased in preterm hAECs. This suggests that the gestational age of the hAEC donor can influence differential response of hAECs towards an inflammatory stimulus. Given our current understanding of how stem cells and stem-like cells can respond to environmental priming [26, 27], the findings from this study may have implications on the application of hAECs collected from donors across different gestational ages.

While hAECs have been reported to secrete angiogenic factors* in vitro* [11], this is the first time that* FOXC1* expression by hAECs has been reported. FOXC1 is a transcription factor involved in regulating vascular development. It is critical for pericyte regulation of vascular development in the mouse fetal brain [28] and is essential for maintaining the integrity of basement membrane and decreasing vascular permeability in zebrafish [29]. FOXC1 also reportedly regulates proangiogenic factors such as matrix metalloproteinases (MMPs) as well as VEGF receptor-ligand signalling [30, 31]. Although VEGFR1 has higher affinity for VEGF, it has a much weaker kinase activity and is thus unable to generate a proangiogenic effect [32]. Indeed VEGFR1 can competitively inhibit the proangiogenic effects of VEGFR2 and thus can be considered as being “antiangiogenic” [33]. In contrast, both PDGFR*α* and PDGFR*β*, the two subunits of PDGF receptors, can bind to PDGFB and contribute to angiogenesis. Although there is no clear separation between the operating mechanisms of the two receptor subunits, PDGFR*β* plays a major role in angiogenic processes in huVECs [34]. PDGFR*β* is required for the stabilisation of newly formed blood vessels, while PDGFR*α* works in significant synergy [35]. PDGFR*β* supports pericyte/endothelial cell interactions and pericyte formation by mediating VEGF expression [36]. Increased PDGFR-kinase activity is associated with elevated expression of VEGFA and VEGFR2, acting directly on endothelial cells and resulting in increased vessel formation [37]. Tie-2 is also an endothelium-specific receptor. When ANGPT1 binds to and activates Tie-2, it induces vascular stabilisation and triggers angiogenesis. The Ang-1/Tie-2 system stabilises preexisting vessels and accelerates angiogenesis when cell-cell adhesion is disrupted [38].

Given the transcriptional changes to proangiogenic ligands in hAECs, we next assessed changes to their receptors in huVECs in a coculture system. When huVECs were cocultured with term hAECs under basal conditions, we saw an increase in* PDGFRα* transcription but a reduction in* Tie-2*. In the presence of IFN*γ* and TNF*α*, however, we observed a reduction in* Tie-2* as well as* FOXC1.* When huVECs were cocultured with preterm hAECs in the presence of IFN*γ* and TNF*α*, we observed an increase in* PDGFRα and PDGFRβ* but a reduction in* FOXC1* gene transcription. These findings indicate that the relationship between hAECs and endothelial cells is complex, and multiple receptor-ligand signalling pathways are likely to be activated during hAEC-mediated angiogenesis. For example, MSCs exert proangiogenic effects through VEGF during wound healing [26] and yet they suppress neovascularisation in chemically injured rat corneas [27].

Next we employed two animal models of lung injury to determine how hAECs affect angiogenesis* in vivo* and if these effects are dependent on the underlying pathology of the injury. Bleomycin-induced pulmonary fibrosis is associated with neovascularisation, where the imbalance of pro- and antiangiogenic mediators is a perpetuator of lung fibrosis [39]. Indeed inhibitors of angiogenesis such as intedanib have been investigated as treatments for lung fibrosis [40]. In our current study we observed that excessive angiogenesis was inhibited in bleomycin challenged mice after the administration of term but not preterm hAECs. This concurs with our previous findings where we showed that term but not preterm hAECs mitigated bleomycin-induced lung injury [13]. In keeping with our* in vitro* findings where we showed that the expression levels of* Tie-2* and* FoxC1* in huVECs were reduced following coculture with term hAECs in the presence of IFN*γ* and TNF*α*, hAECs may reduce excessive angiogenesis by downregulating angiogenic factor receptors on the endothelial cells.

Since there were only observable differences in the vWF staining following term and hAEC treatment in the bleomycin model, we next applied only term hAECs to the hyperoxia neonatal lung injury model. Alveolarisation and angiogenesis are essential for normal lung development and the blood vessels in the lung promote normal alveolar development and contribute to maintenance of alveolar structure [41]. We observed that the number of smaller blood vessels was reduced following hyperoxia and this was restored by hAEC treatment, indicating that hAEC treatment may have affected angiogenesis in conjunction with the reversal of alveolar simplification as previously reported [5]. Our findings from the two lung injury models suggest that angiogenesis may be the driving force for hAEC-mediated lung repair; however, it is equally important to appreciate that angiogenic factors such as VEGF and PDGFB can also act as proinflammatory cytokines rather than solely as angiogenic factors during lung injury [4, 6]. In particular, the process of angiogenesis can perpetuate inflammation depending on concurrent events such as enhanced adhesion and increased endothelial permeability [42].

In conclusion, we showed that angiogenesis may be one of the mechanisms through which hAECs augment lung repair. We also report on differential angiogenic potentials between term and preterm hAECs, which may have profound implications on donor sourcing and clinical applications of these cells. Further, we show that inflammatory cytokines such as IFN*γ* and TNF*α* can impact the angiogenic properties of hAECs and these findings may extend to other stem cells and stem-like cells as well as other mechanisms of action. Given that stem cell priming has become a topical discussion point of late, the impact of microenvironmental cues on stem cell functionality should be considered when identifying optimal times of cell administration.

## Figures and Tables

**Table 1:** Gestational age of both term and preterm birth.

	Gestational age (d)			Complications
	Minimum	Maximum	Mean ± SEM	
Preterm birth (*n* = 9)	196	257	230.4 ± 7.37	IUGR (*n* = 5); PE (*n* = 4)
Term birth (*n* = 16)	261	277	268.3 ± 1.03	Nil

**Table 2:** Primer sequences and annealing temperatures.

Gene	Primer sequence	Annealing temperature
VEGFA	Fwd: CTACCTCCACCATGCCAAGTG	60°C
	Rev: TGATTCTGCCCTCCTCCTTCT	

PDGFB	Fwd: AATGGTCACCCGAGTTTGG	60°C
	Rev: CTGGCATGCAAGTGTGAGAC	

ANGPT1	Fwd: CCTGATCTTACACGGTGCTGATT	60°C
	Rev: GTCCCGCAGTATAGAACATTCCA	

VEGFR1	Fwd: CGGGGATTTCACTGTACATCT	60°C
	Rev: AAGCAAACCACACTGGCTTC	

VEGFR2	Fwd: CCCTGCCGTGTTGAAGAGTT	60°C
	Rev: GGACAGGGGGAAGAACAAAA	

PDGFR*α*	Fwd: AGCTGGCAGAGGATTAGGCT	60°C
	Rev: CTCCATGTGTGGGACATTCA	

PDGFR*β*	Fwd: CAGGAGAGACAGCAACAGCA	60°C
	Rev: TGTCCAGAGCCTGGAACTGT	

Tie-2	Fwd: AGTCTTATGTGTTCTGTCTCCCTGACC	60°C
	Rev: TCATCCTCGGTATGCCTTCTCTCTCAC	

FOXC1	Fwd: ACCTTGACGAAGCACTCGTT	60°C
	Rev: CGGCATCTACCAGTTCATCA	

## Abbreviations

huVECs: Human umbilical vein endothelial cells
hAECs: Human amnion epithelial cells
TNF*α*: Tumor necrosis factor *α*
IFN*γ*: Interferon *γ*
VEGFA: Vascular endothelial growth factor
PDGFB: Platelet derived growth factor B
ANGPT1: Angiogenin-1
Foxc1: Forkhead box (Fox) transcription factor c1
VEGFR1 and VEGFR2: Vascular endothelial growth factor receptors 1 and 2
PDGFR*α* and PDGFR*β*: Platelet derived growth factor receptors alpha and beta
vWF: von Willebrand factor.
